# An antibody raised against a pathogenic serpin variant induces mutant-like behaviour in the wild-type protein

**DOI:** 10.1042/BJ20141569

**Published:** 2015-05-05

**Authors:** James A. Irving, Elena Miranda, Imran Haq, Juan Perez, Vadim R. Kotov, Sarah V. Faull, Neda Motamedi-Shad, David A. Lomas

**Affiliations:** *Wolfson Institute for Biomedical Research, University College London, Gower Street, London WC1E 6BN, U.K.; †Department of Biology and Biotechnologies ‘Charles Darwin’ and Pasteur Institute-Cenci Bolognetti Foundation, Sapienza University of Rome, Piazzale Aldo Moro 5, 00185 Rome, Italy; ‡Department of Cell Biology, Genetics and Animal Physiology, University of Malaga, Campus de Teatinos, 29071 Malaga, Spain; §Department of Pharmacology, Robert Wood Johnson Medical School, Rutgers University, 675 Hoes Lane Piscataway, NJ 08854, U.S.A.; ∥Cambridge Institute for Medical Research, University of Cambridge, Hills Road, Cambridge CB2 0XY, U.K.

**Keywords:** aggregation, antibody, α_1_-antitrypsin, polymerization, protein stability, AF488, Alexa Fluor 488 dye, AF594, Alexa Fluor 594 dye, AT, α_1_-antitrypsin, ER, endoplasmic reticulum, H/D exchange, hydrogen/deuterium exchange, HRP, horseradish peroxidase, mAb, monoclonal antibody, PBS, phosphate-buffered saline

## Abstract

A monoclonal antibody (mAb) that binds to a transient intermediate may act as a catalyst for the corresponding reaction; here we show this principle can extend on a macro molecular scale to the induction of mutant-like oligomerization in a wild-type protein. Using the common pathogenic E342K (Z) variant of α_1_-antitrypsin as antigen–whose native state is susceptible to the formation of a proto-oligomeric intermediate–we have produced a mAb (5E3) that increases the rate of oligomerization of the wild-type (M) variant. Employing ELISA, gel shift, thermal stability and FRET time-course experiments, we show that mAb_5E3_ does not bind to the native state of α_1_-antitrypsin, but recognizes a cryptic epitope in the vicinity of the post-helix A loop and strand 4C that is revealed upon transition to the polymerization intermediate, and which persists in the ensuing oligomer. This epitope is not shared by loop-inserted monomeric conformations. We show the increased amenity to polymerization by either the pathogenic E342K mutation or the binding of mAb_5E3_ occurs without affecting the energetic barrier to polymerization. As mAb_5E3_ also does not alter the relative stability of the monomer to intermediate, it acts in a manner similar to the E342K mutant, by facilitating the conformational interchange between these two states.

## Abstract 2

We show that a monoclonal antibody can act as a ‘molecular template’ in aberrant protein oligomerization, and the transient intermediate of α_1_-antitrypsin, a key to the molecular mechanism of disease pathogenesis, expresses a cryptic epitope also present in the oligomer.

## INTRODUCTION

α_1_-Antitrypsin (*SERPINA1*) is an acute-phase glycoprotein, secreted by hepatocytes and abundant in the plasma. A member of the serpin superfamily, the primary role of α_1_-antitrypsin is the inactivation of neutrophil elastase in the lung. The pathophysiological importance of this protein is underscored by individuals harbouring loss-of-function mutations, who have a pre-disposition to emphysema [1], and those who exhibit a gain-of-function phenotype: the formation of recalcitrant protein deposits in the endoplasmic reticulum (ER) of the cell of synthesis. These inclusions atypically fail to induce the ER unfolded protein response [2,3], but cause distension of the organelle, interfere with protein diffusion [4] and can lead to liver cirrhosis [5]. Currently, the only commonly practised clinical interventions are replacement therapy, which is both costly and controversial as to its efficacy [6], and liver transplantation, where end-stage liver disease has ensued [5].

The most common pathological variant associated with α_1_-antitrypsin deficiency is the Z allele (E342K), which is present in ∼4% of Northern Europeans. This mutation has been shown to facilitate the transition between the native state and a conformation that is prone to the formation of ordered aggregates, termed polymers [7]. Biophysical approaches have indicated that polymer formation proceeds from monomer through several unimolecular ‘activation’ steps [8,9]. The pathway culminates in a bimolecular association that, being essentially irreversible, renders the process under kinetic control [10]. Polymers isolated from patients homozygous for the Z allelle have an unbranched beads-on-a-string morphology [11,12], and several hypotheses as to the underlying inter-molecular linkage have been advanced based on biophysical [11–14] and crystallographic [15] evidence. Chief among these are the A-sheet model [11], the β-hairpin model [14] and the C-terminal model [15]. Although it remains to be demonstrated which of these polymer states reflects those present in patient populations, these disparate proposals suggest that under certain conditions α_1_-antitrypsin can access multiple end-point polymer states. Nevertheless, the inherent stability and size heterogeneity of the final polymer form renders it a non-ideal target for therapeutic interventions to mitigate the intra-hepatic formation or deposition of polymers. Instead, manipulation of the polymerization pathway itself represents a more tractable approach [16]. However, a detailed molecular understanding of this pathway requires tools for probing its intermediates.

Many examples exist of antibodies and related molecules that exhibit conformation-specific recognition of targets such as transthyretin [17], superoxide dismutase [18] and serpins, including plasminogen activator inhibitor-1 [19] and α_1_-antitrypsin [20]. In addition, some antibodies are known to alter the behaviour of their cognate antigen, such as stabilizing against [21] and inducing [22] conformational change. From a screen developed to identify monoclonal antibodies (mAbs) that alter the rate of polymerization of α_1_-antitrypsin, we identified one, mAb_5E3_, that demonstrated both conformational specificity and antigen-altering activity. This molecule was found to recognize a cryptic epitope revealed in the transition to a polymerization intermediate, and that persists in the ensuing polymer. Both the pathogenic E342K variant purified from patient plasma, and the wild-type M variant in the presence of the mAb, showed a similar polymerization activation energy to wild-type protein alone. Thus, this mAb increases conformational flux between native and activated forms without affecting their relative thermodynamic stabilities and without interfering with subsequent oligomerization. These data show that an antibody raised against an unstable mutant can act as a molecular template to induce aberrant behaviour in a wild-type protein.

## EXPERIMENTAL

### Preparation of α_1_-antitrypsin

M (AT_WT_) and Z (AT_E342K_) α_1_-antitrypsin were isolated from plasma of PI*MM and PI*ZZ α_1_-antitrypsin homozygotes as detailed previously [12]. Control AT_WT_ or AT_E342K_ heat-induced polymers were prepared by heating the monomeric protein (0.2 mg·ml^−1^) at 60°C in phosphate-buffered saline (PBS) (pH 7.4) for 3 h, and confirmed by non-denaturing PAGE. Denaturant-induced polymers were generated using 3M guanidinium chloride in PBS for 48 h at room temperature. Variants of recombinant α_1_-antitrypsin were generated by site-directed mutagenesis, expressed in *Escherichia coli* XL-1 Blue, and purified to homogeneity by affinity chromatography using nickel-nitrilotriacetate (NTA)–S sepharose (Qiagen) and Q Sepharose (GE Healthcare Life Sciences) as detailed previously [9,10]. Fab fragments were generated using a commercial papain-based kit according to the manufacturers' instructions (Thermo Scientific).

### Production and screening of antibodies that alter polymerization of AT_E342K_

Balb/c mice were immunized with monomeric AT_E342K_, and spleen cells were harvested and fused with myeloma cells. The supernatants of the resulting hybridoma clones were screened first by antigen-mediated ELISA using purified AT_E342K_ monomer as the antigen; those of selected clones were then heated at 45°C for 45 h in the presence of 20 μg·ml^−1^ AT_E342K_, and the consequent polymers were quantified using an ELISA with the polymer-specific antibody mAb_2C1_ and a non-conformation sensitive antibody, mAb_9C5_ [20]. To verify whether identified antibodies were interfering with the assay itself, a competitive ELISA was performed in which the plate was coated with polymer-specific mAb_2C1_, loaded with excess polymer antigen, and different dilutions of cell culture supernatants were applied. Detection was by horseradish peroxidase (HRP)-conjugated mAb_9C5_. The clone showing increased polymerization levels with respect to a media-only control was sub-cloned by limited dilution, expanded as a cell line, and characterized by antigen-mediated, competitive, polyclonal sandwich and monoclonal sandwich ELISA.

### Antigen and sandwich ELISA

The protocols used have been described previously [20]. Plates were coated overnight with antigen-purified rabbit polyclonal anti-α_1_-antitrypsin antibody at 2 μg·ml^−1^ in PBS for sandwich ELISA, or purified AT_E342K_ in PBS for antigen-mediated ELISA. Following this they were washed with 0.9% (w/v) sodium chloride and 0.05% (v/v) Tween 20 and blocked for 2 h with 300 μl per well of blocking buffer (PBS, 0.25% BSA, 0.05% Tween 20 and 0.025% sodium azide). For sandwich ELISA, samples were diluted in blocking buffer to a final volume of 50 μl, added to the plate and incubated for 2 h. After washing, for both types of ELISA the wells were incubated for 2 h with the primary mAbs (mAb_5E3_ or polymer-specific mAb_2C1_) at a concentration of 1 μg·ml^−1^ diluted in blocking buffer, the wells were washed again and rabbit anti-mouse HRP-conjugated antibody (Sigma) was added for 1 h. After removing residual secondary antibody, the reaction was developed for 10 min with 3,3′,5,5′-tetramethylbenzidine (TMB) substrate solution (Sigma–Aldrich), stopped with 1 M H_2_SO_4_, and an endpoint measurement of HRP activity at 450 nm was made using a SpectraMax M2 plate reader (Molecular Devices). All steps were performed at room temperature.

### Native thermal stability assays

Experiments, using the SYPRO Orange fluorescent reporter (Life Technologies) were performed as detailed previously [10], and the temperature midpoint was determined by application of an equation describing thermal denaturation to the data [23,24]:

$$F_{T}=F_{N}+m_{N}\left(T-T_{m}\right)+\left(F_{I}+m_{I}\left(T-T_{m}\right)\right)\frac{e^{C\left(\frac{1}{T_{m}}-\frac{1}{T}\right)}}{1+e^{C\left(\frac{1}{T_{m}}-\frac{1}{T}\right)}}$$

where *F*_T_ is the fluorescence at temperature *T*, *T*_m_ is the transition midpoint temperature, *F*_N_ and *F*_I_ are the fluorescence of the native and intermediate states, *m*_N_ and *m*_I_ describe their temperature dependence around temperature *T*_m_, and *C*=Δ*H*_m_/*R*, the enthalpy of unfolding at *T*_m_ divided by the gas constant. As the reaction is not at equilibrium [10] the optimized value of Δ*H*_m_ was not used.

### Thiol labelling of plasma α_1_-antitrypsin

α_1_-Antitrypsin has a single endogenous surface-exposed cysteine residue (Cys^232^) that is readily conjugated with thiol-reactive probes [8,25]. Variants of α_1_-antitrypsin were reduced using 20 mM β-mercaptoethanol, buffer exchanged into PBS, and incubated with a 10-fold molar excess of Alexa Fluor 488 (AF488) maleimide or Alexa Fluor 594 (AF594) maleimide (Life Technologies) overnight at 4°C with reaction quenching using 5 mM cysteine. Application to, and elution from, a 1 ml HiTrap Q Sepharose column was used to remove remaining free label.

### FRET-based polymerization assays

α_1_-Antitrypsin, labelled with fluorophores at residue Cys^232^, was diluted to 0.1 mg·ml^−1^ in PBS in the presence and absence of 0.45 mg·ml^−1^ antibody and a final volume of 10 μl. Polymerization was reported by an increase over time in FRET between the AF488 donor dye (excitation at 470 nm) and the AF594 acceptor dye (emission recorded at 605±15 nm) upon heating in a Realplex^4^ quantitative PCR instrument (Eppendorf). The Förster radius of this pair is ∼60 Å (1 Å=0.1 nm) [26], which makes them useful at distances predicted by the different models of polymerization to arise between monomers in a repeating polymer [14,15,25,27,28]. The increase in FRET signal was found to be satisfactorily fitted by a single or double sigmoidal curve in Prism (GraphPad):

$$F_{t}=H\times t^{2}\times \left(\frac{w}{\left(t_{0.5,1}^{2}+t^{2}\right)}+\frac{\left(1-w\right)}{\left(t_{0.5,2}^{2}+t^{2}\right)}\right)+L$$

where *F_t_* is the fluorescence at time *t*, *H* is the dynamic range of the curve, *L* is the baseline, *w* is the fraction contribution to the signal by the curve with a half-time of *t*_0.5,1_ and (1−*w*) is the fraction contribution to the signal by the curve with a half-time of *t*_0.5,2_. This equation permitted numerical calculation by the software of the overall time to half-maximal fluorescence intensity.

### Gel-based experiments

Bis-Tris non-denaturing PAGE (3–12% (w/v) acrylamide) (Life Technologies) was used to determine the oligomeric state of α_1_-antitrypsin and perform gel shift experiments, with 2–4 μg of protein per lane for staining with Coomassie Blue and 0.05–2 μg for fluorescence detection using a UV transilluminator. This gel system was also used for mobility shift experiments; antibodies cause a decreased rate of α_1_-antitrypsin migration due both to the increased size of the antibody–antigen complex and an elevated overall isoelectric point. Densitometry was performed using ImageJ [29].

## RESULTS

### Identification of a monoclonal antibody that increases the rate of polymerization

**Figure 1:**
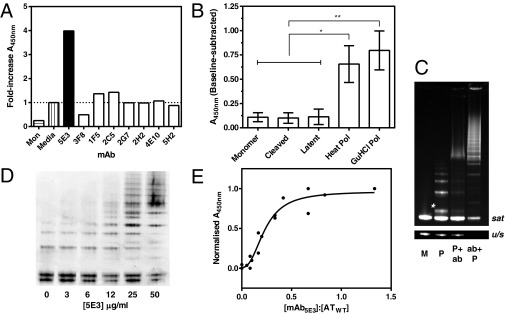
Screens for mAbs that alter polymerization, and the specificity of mAb_5E3_ (**A**) Cell culture supernatants of hybridoma clones including 5E3 (black bar) were incubated at 45°C for 45 h with AT_E342K_ at 20 μg·ml^−1^, and polymer levels were quantified by sandwich ELISA using plates coated with the polymer-specific mAb_2C1_ and detection by mAb_9C5_ conjugated to HRP. Values shown are fold-increases in polymer levels with respect to a media-only control (shaded bar). (**B**) A sandwich ELISA performed using plates coated with anti-α_1_-antitrypsin polyclonal antibody, bound for 2 h with serially diluted conformers of AT_WT_, and detection by mAb_5E3_ at 1 μg·ml^−1^ and an HRP-conjugated secondary antibody. After baseline subtraction the data at different dilutions were fitted to a four-parameter logistic function and the values at a total antigen load of 2.5 ng recorded (mean±S.E.M. for four independent experiments). A one-way ANOVA using Bonferroni's correction indicated a significant difference between polymers and monomers (*P<0.05; **P<0.01). (**C**) Gel shift experiment using AF488/AF594-labelled AT_WT_ protein separated by 3–12% (w/v) non-denaturing PAGE: ‘M’, unheated monomer; ‘P’, monomer heated at 53°C for 12 h; ‘P+ab’, the same heated monomer mixed with a 1.5-fold molar excess of mAb_5E3_ 5 min prior to electrophoresis; and ‘ab+P’, monomer incubated for 5 min with mAb_5E3_ prior to heating at 53°C for 12 h. Detection was by fluorescence using a UV transilluminator. The upper panel has deliberately been overexposed (‘*sat*’) to highlight the oligomeric region of the gel; the lower panel shows the affected monomer region at a lower intensity (‘*u/s*’). The asterisk indicates a species with a migration intermediate between monomer and dimer that appears to represent a polymerization intermediate [33]. (**D**) Purified AT_E342K_ was incubated at 20 μg·ml^−1^ for 45 h at 45°C in the presence of 0–50 μg·ml^−1^ mAb_5E3_ and the resulting polymers were quantified by silver-stained non-denaturing PAGE. (**E**) Combined data from mAb_2C1_ ELISA following incubation of 20 μg·ml^−1^ or 100 μg·ml^−1^ AT_E342K_ for 45 h at 45°C with a 0–1.33-fold molar ratio of mAb_5E3_. The resulting absorbance readings from each of three experiments were normalized to lie between 0 and 1, and the abscissa shows the molar ratio of mAb_5E3_ to α_1_-antitrypsin concentration. The curve reflects a four-parameter logistic equation; a half-maximal effect was observed at a molar stoichiometry of 0.23±0.03.

We set out to identify, from a panel of mAbs raised against an oligomerization-prone variant of α_1_-antitrypsin, one that could accelerate polymerization, and determine whether it could induce comparable behaviour in the wild-type protein. Standard protocols were followed to identify hybridomas expressing mouse monoclonal antibodies against purified Z α_1_-antitrypsin (AT_E342K_) [20,30]. Aliquots of cell culture supernatant from these hybridomas were mixed with AT_E342K_, heated at 45°C for 45 h, and polymer levels were quantified by a sandwich ELISA using plates coated with the polymer-specific mAb_2C1_ and detection of bound polymers with conformation-insensitive mAb_9C5_–HRP [20]. One clone, 5E3, increased the level of polymer ∼4-fold with respect to a media-only control (Figure 1A), and the effect was confirmed using purified antibody in a sandwich ELISA (Supplementary Figure S1A) and interference assay (Supplementary Figure S1B). Subsequent experiments made use of purified mAb_5E3_.

### mAb_5E3_ shows specificity for heat- and denaturant-induced polymers

Polymerization is a kinetically controlled process, and comprises at least one unimolecular activation step and a bimolecular association step [9]:

$$\begin{array}{c} M↔M^{*}\\ M^{*}+M^{*}\to P \end{array}$$

The consequence of this scheme is that the reaction does not reach equilibrium; there is a positive flux of monomer incorporated into oligomers in a manner influenced by protein concentration and the activation energy barrier to oligomer formation. Consequently, an increased polymerization rate could be achieved through changes in thermodynamic stability of different components of the pathway or a reduction in energy required to transition from one state to the next [7]. Thus, any conformational selectivity could play a role in the apparent polymerization-enhancing activity of mAb_5E3_.

Experiments were performed to characterize any preferential binding of mAb_5E3_ to different conformations of α_1_-antitrypsin. A Western blot of heated AT_E342K_ samples indicated that mAb_5E3_ is able to recognize heat-induced polymer (Supplementary Figure S1C). However, it is unlikely that binding to the terminal polymer form would increase the rate of polymerization, as this form is itself extremely stable [11,31] with at best marginal dissociation reported [32]. Recognition of different monomer conformations and heat- and guanidium chloride-induced polymers were compared by ELISA (Figure 1B). The three main proposed models for the structure of the pathological polymer countenance a monomeric unit that is in a loop-inserted conformation, consistent to varying degrees with the cleaved and latent states of the protein. However, these results revealed a distinct preference for polymer over monomer, and in contrast with mAb_2C1_ [20], no clear selectivity between denaturant- and heat-induced polymer forms.

Monoclonal antibodies migrate more slowly in non-denaturing gels than α_1_-antitrypsin due to their larger size and higher isoelectric point. We exploited this in performing a gel shift experiment using AT_WT_ that had been fluorescently labelled at Cys^232^ with AF488 and AF594 dyes (Figure 1C). M α_1_-antitrypsin (AT_WT_) was polymerized at 53°C for 12 h, either in the presence of a 1.5-fold molar excess of mAb_5E3_ (lane 4) or alone (lane 2) and pre-made polymers were also mixed with the same proportion of antibody 5 min prior to electrophoresis by non-denaturing PAGE (lane 3). A comparison of lanes 2 and 3 of the gel revealed sequestration of the oligomeric material by the antibody into a slower-migrating species, without a reduction in intensity of the monomer band. Interestingly, a band interposed between monomer and dimer was also bound by the antibody; such a species has been identified previously as a hallmark of the polymerization intermediate [33]. AT_WT_ polymerized in the presence of mAb_5E3_ demonstrated an increased quantity of higher-order material and a corresponding decrease in the amount of monomer left at the end of the incubation, providing a secondary confirmation of the polymerization-enhancing activity.

### mAb_5E3_ increases polymerization in a dose-dependent manner

The experiments in Figures 1(B) and 1(C) indicate that mAb_5E3_ is able to form a stable complex with polymer and possibly an intermediate species as well. If a stable interaction is the basis of its activity, then the antibody would be expected to act in a dose-dependent fashion at a comparable range of concentrations to α_1_-antitrypsin. A contrasting scenario would be one in which the antibody acts as a catalyst by transiently binding and stabilizing a transition state on the polymerization pathway, which could occur at markedly lower molar ratios. Aliquots of AT_E342K_ were incubated with different concentrations of mAb_5E3_ at 45°C for 45 h, and the presence of polymers was identified by non-denaturing PAGE (Figure 1D) and quantified by ELISA using mAb_2C1_ (Figure 1E). Both techniques were found to report a dose-dependent increase in polymers at higher mAb_5E3_ concentrations with respect to the control, with the latter data fitted to a four-parameter logistic equation yielding an EC_50_ value of 0.23±0.03. As IgG are divalent, this is equivalent to a binding-site ratio of 0.46:1 required to achieve a half-maximal effect. Thus, mAb_5E3_ most likely operates by binding in a near-stoichiometric fashion to a component of the polymerization pathway following activation of the monomer species.

### mAb_5E3_ has no effect on the equilibrium between native and intermediate forms

**Figure 2:**
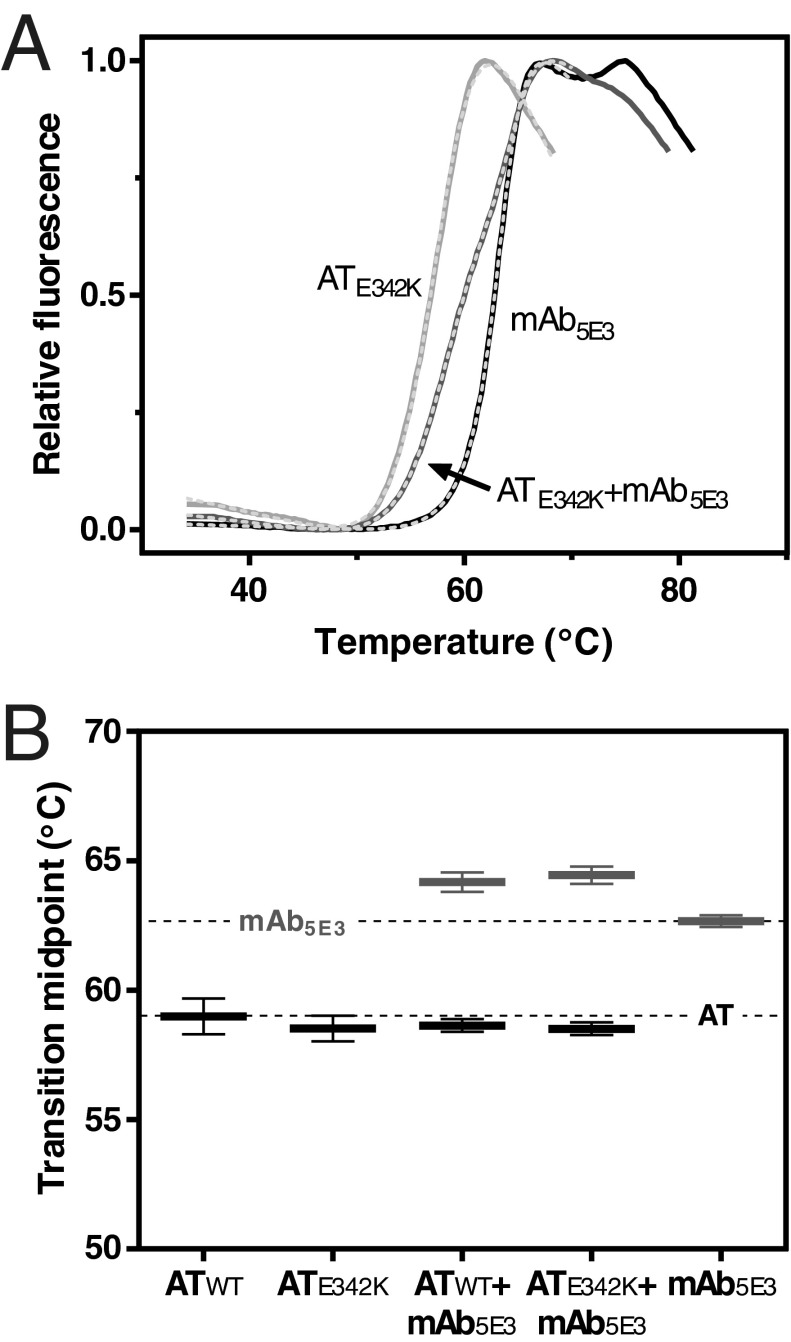
Effect of mAb_5E3_ on the transition to a polymerization intermediate (**A**) A representative SYPRO Orange thermal shift experiment using 0.1 mg·ml^−1^ AT_E342K_ in the presence (medium grey) and absence (light grey) of a 1.5-fold molar excess of mAb_5E3_ (black). Samples were heated from 25°C to 95°C at a rate of 1°C·min^−1^. The fluorescence of SYPRO Orange with α_1_-antitrypsin reports transition to a polymerization intermediate [10]. Transition midpoints were inferred for each protein by non-linear regression, shown as a white overlaid dashed line, as described in the text. (**B**) Thermal midpoints of denaturation were calculated for 0.1 mg·ml^−1^ AT_WT_, AT_E342K_ and a 1.5-fold molar concentration of mAb_5E3_ alone or in combination. Data were fitted by a single or double two-state unfolding equation, used to de-convolute the contribution from mAb_5E3_ (grey) and α_1_-antitrypsin (black) components. The temperature gradient was 1°C·min^−1^. Error bars reflect ±SEM of at least four individual experiments.

Thermal shift experiments using SYPRO Orange report the transition from the α_1_-antitrypsin native state to a pre-polymer intermediate [10], and thus should indicate whether the antibody plays an active role in this process. From normalized thermograms (Figure 2A), it was possible to obtain thermal midpoint temperatures (*T*_m_) of the individual components alone or in combination by non-linear regression of a double two-state unfolding equation (Figure 2B). It was observed that the presence of the antibody had a negligible effect on the transition midpoint of α_1_-antitrypsin, but a slight stabilization (by 1.7±0.3°C) of the antibody. As mAb_5E3_ unfolding occurs at a higher temperature than the conversion of α_1_-antitrypsin to intermediate, this is probably to be the result of stabilization due to complex formation either with the intermediate or a later-stage species such as polymer.

### Progress curves report a mAb_5E3_-induced increased rate of polymerization

**Figure 3:**
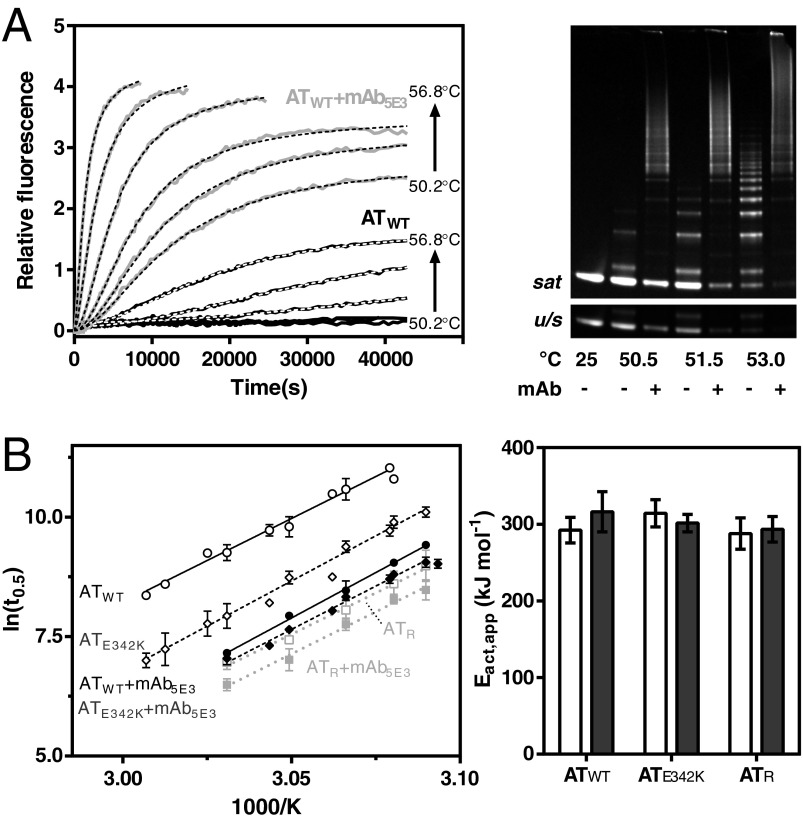
mAb_5E3_ increases the rate of α_1_-antitrypsin polymerization (**A**) Left panel: an equimolar population of AT_WT_ labelled either with a donor (AF488) fluorophore or an acceptor (AF594) fluorophore was incubated at 0.1 mg·ml^−1^, and polymerization was monitored as an increase in FRET. Curves representing samples incubated at different temperatures in the presence (grey lines) and absence (black lines) of 0.45 mg·ml^−1^ mAb_5E3_ are shown. Overlaid dashed lines (black and white, respectively) show the curves of best fit. Right panel: 0.1 mg·ml^−1^ AT_WT_ labelled with AF488 and AF594 maleimide heated at various temperatures for 12 h in the absence and presence of a 1.5-fold molar ratio of mAb_5E3_, separated by non-denaturing PAGE and visualized using a UV transilluminator. The upper panel has been overexposed (‘*sat*’) and the lower panel shows the affected monomer region at a lower intensity (‘*u/s*’). (**B**) Left panel: an Arrhenius analysis of polymerization half-times as determined from FRET-based progress curve recorded at temperatures of 50–60°C for 0.1 mg·ml^−1^ AT_WT_ (black, unbroken lines, circles), AT_E342K_ (medium grey, dashed lines, diamonds) and AT_R_ (light grey, dotted lines, squares) in the presence (shaded symbols) or absence (open symbols) of a 1.5-fold molar concentration of mAb_5E3_. The data arise from at least four independent experiments and are means ± S.D. of two or more observations at a given temperature. Right panel: the apparent activation energy (mean±S.E.M.) for the polymerization of α_1_-antitrypsin was calculated from the slopes of linear regressions. There was no significant difference between the different forms of α_1_-antitrypsin in the presence and absence of mAb_5E3_, with values of ∼300 kJ·mol^−1^.

AT_WT_ was labelled via the endogenous cysteine residue at position 232 with AF488 maleimide and AF594 maleimide dyes. Despite stark differences, the various polymer models broadly agree that fluorophores of adjacent monomers would be brought within a distance of less than 80 Å in the polymer chain [8,14,15,27,28]. Thus, temperature-induced polymerization could be monitored based on the increase in FRET between this pair of probes–whose Förster radius is 60 Å [26]–with excitation of AF488 at 470 nm and emission recorded from AF594 at 605 nm (±15 nm). Figure 3(A, left panel) shows progress curves recorded for AT_WT_ at temperatures between 50.2°C and 56.8°C. A clear stimulatory effect was seen in the presence of the antibody, even at temperatures otherwise with no detectable polymerization, highlighted by a non-denaturing gel (Figure 3A, right panel), which showed at the conclusion of the experiment a depleted monomer and substantial amount of sequestered polymer in the presence of mAb_5E3_ with respect to antibody-free controls. This acceleration was not due simply to a template mechanism by the antibody, as in dose–response experiments characteristics of the FRET curves were found to be monophasic with respect to concentration, rather than bell-shaped (Supplementary Figures S1D–S1E).

### mAb_5E3_ does not affect the polymerization activation energy

In the typical range of temperatures used to induce polymerization, the rate at which α_1_-antitrypsin polymers form shows an Arrhenius-type temperature dependence [9,10]. AT_E342K_ and recombinant wild-type (AT_R_) were fluorescently labelled in the same manner as AT_WT_. Both have an inherently increased rate of polymerization with respect to the wild-type protein: AT_E342K_ has a decreased kinetic stability against conversion to the polymerization intermediate [7], whereas, due to expression in *E. coli*, AT_R_ lacks the three glycan chains present in plasma-derived material (on aparagine residues at positions 46, 83 and 247), which are associated with maintaining a compact monomeric state [34]. Half-times of polymerization were derived using an empirical sigmoidal, or double-sigmoidal, function from FRET progress curves of AT_WT_, AT_E342K_ and AT_R_ in the presence and absence of mAb_5E3_ at temperatures between 50°C and 60°C (Figure 3B, left panel). Consistent with its well-characterized tendency to form polymers, polymerization half-times for AT_E342K_ alone were lower than for AT_WT_. The rate of polymerization of both variants was substantially increased in the presence of mAb_5E3_, such that the two became almost equivalent in behaviour, as seen in the strikingly similar regression lines. Thus, despite the intrinsic kinetic instability of AT_E342K_ [7], mAb_5E3_ caused a convergence of the response of both variants to thermal challenge. Notably, the rates for the glycosylated plasma proteins in the presence of antibody are comparable to AT_R_ (expressed in *E. coli* and therefore non-glycosylated) without antibody. The presence of mAb_5E3_ caused a detectable, but less pronounced, acceleration of AT_R_ polymerization.

Apparent activation energy (*E*_act,app_) values were calculated from the linear regressions (Table 1). At ∼300 kJ·mol^−1^–consistent with previous calculations for wild-type recombinant α_1_-antitrypsin [10]–no significant difference was observed between AT_E342K_, AT_WT_ or AT_R_ either in the presence or in the absence of antibody (Figure 3B, right panel). This analysis shows that neither the Z mutation, or the absence of glycan chains from asparagine residues at positions 46, 83 and 247, nor the presence of mAb_5E3_ affects the overall energetic barrier to polymerization.

**Table 1:** Polymerization of α_1_-antitrypsin variants in the presence and absence of mAb_5E3_ A summary of the data in Figure 3(B), showing the times taken to reach a half-maximal polymerization FRET signal at 53°C, and the calculated apparent activation energy of the reaction (*E*_act_). Results are means±S.E.M.

Sample	*t*_0.5_ at 53°C (s)	*E*_act_ (kJ·mol^−1^)
AT_WT_	37.8(±2.3)×10^3^	292±17
AT_WT_ + mAb_5E3_	4.9(±0.3)×10^3^	316±26
AT_E342K_	10.7(±0.7)×10^3^	314±17
AT_E342K_ + mAb_5E3_	3.8(±0.1)×10^3^	301±11
AT_R_	3.3(±0.2)×10^3^	287±20
AT_R_ + mAb_5E3_	2.2(±0.1)×10^3^	293±17

### A monovalent Fab fragment increases the rate of polymerization of AT_WT_

**Figure 4:**
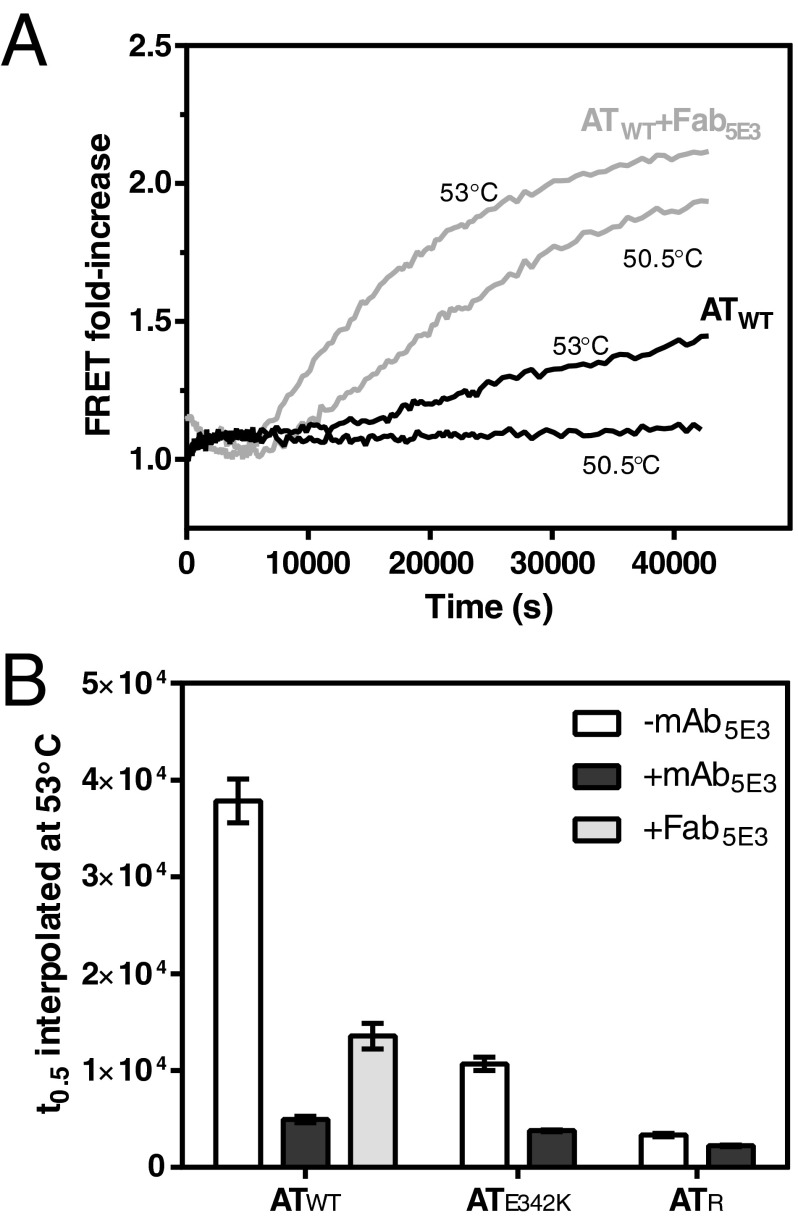
Increased rate of α_1_-antitrypsin polymerization in the presence of Fab_5E3_ (**A**) Representative FRET-based progress curves showing the polymerization of AT_WT_ in the presence (grey lines) and absence (black lines) of a 1.5-fold concentration of Fab_5E3_. (**B**) Calculated polymerization half-times (means±S.E.M.) at a reference temperature, 53°C, from at least nine independent data points.

To determine the extent to which divalent binding by mAb_5E3_ contributes to the observed polymerization rates, Fab_5E3_ fragments were generated by digestion with papain and tested on AT_WT_ in FRET-based time-course experiments. As with the full antibody, the induction of polymerization at an otherwise non-favoured temperature was observed (Figure 4A). To quantify this effect, a comparison was made between the half-times of polymerization at a reference temperature (Figure 4B). It was found that the presence of Fab_5E3_ induced a similar rate of polymerization in AT_WT_ to that of AT_E342K_, with a half-time of 13.5(±0.4)×10^3^ s at 53°C. Thus, binding of the mAb_5E3_ epitope by a monovalent ligand resulted in behaviour comparable to that of the antigen against which the antibody had been raised.

### The mAb5E3 epitope is in the vicinity of residues Ser^47^ and Glu^205^

**Figure 5:**
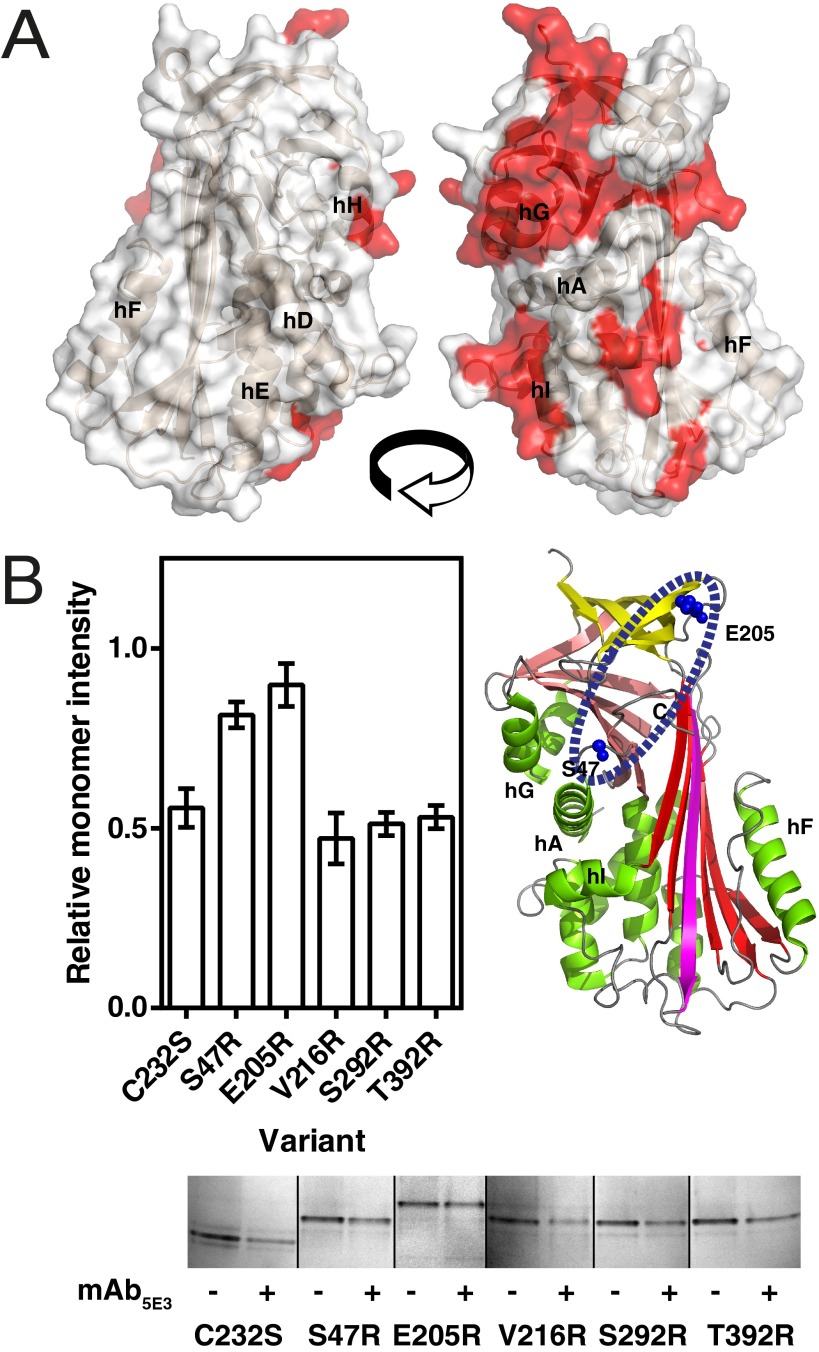
Site of the mAb_5E3_ epitope (**A**) A molecular surface representation of loop-inserted α_1_-antitrypsin (PDB accession 1EZX, chain A), used to approximate the parsimonious conformation of the monomer proposed by the three main models for the terminal polymer [10], with β-sheet A oriented along the Z plane. Peptide fragments associated with transition to a heat- or denaturant-induced polymerization intermediate were identified by reconciling three previous studies [35–37] as described in the text. (**B**) Left panel: several surface-exposed residues that were situated on the face of α_1_-antitrypsin primarily implicated in intermediate formation were mutated to arginine to explore possible involvement in the mAb_5E3_ cryptic epitope. Recombinant protein was heated at 54°C for 30 min in the presence and absence of mAb_5E3_ and residual monomer was quantified using densitometry of non-denaturing PAGE gels (a representative gel is shown in the bottom panel). Results are mean±S.E.M. proportions of residual monomer for each variant in the presence of mAb_5E3_ relative to its absence, calculated from four independent experiments. Right panel: mutations that reduced the effect of mAb_5E3_ on polymerization are shown on a schematic representation of loop-inserted α_1_-antitrypsin. Structure figures were prepared using PyMOL (http://www.pymol.org).

Although polymers induced by heat share a conformational epitope with *ex vivo* material that denaturant-induced polymers do not [33], mAb_5E3_ is able to recognize both forms (Figure 1B). On this basis, an attempt was made to reconcile regions of α_1_-antitrypsin implicated by three previous studies in the transition to intermediate, but that do not change in the transition from intermediate to polymer, based on observations in the literature. Peptide fragments were shortlisted that (a) were among nine exhibiting the greatest difference in hydrogen/deuterium (H/D) exchange between native and intermediate states [35]; (b) were also among nine fragments having the least difference in H/D exchange between intermediate and polymer [35]; and (c) also spanned at least one residue identified by NMR [36] or surface accessibility experiments [37]. The resulting surface map of α_1_-antitrypsin revealed a starkly polarized molecule (Figure 5A).

From this analysis, several initial sites were selected for arginine scanning mutagenesis. Six mutants were expressed in *E. coli* (C232S, S47R, E205R, V216R, S292R and T392R) and purified to homogeneity. Gel-based quantification of recombinant polymer is usually inferred from the loss of monomer following non-denaturing PAGE [38]. These mutants were subjected to a 30-min incubation at 54°C, expected from the AT_R_ polymerization data in Figure 3(B) to yield roughly a 50% depletion of monomer in the presence of mAb_5E3_. The relative amount of residual monomer in the absence and presence of mAb_5E3_ was quantified by non-denaturing PAGE and densitometry in four experiments (Figure 5B, left and bottom panels). Both the S47R or E205R mutants, on the post-helix A loop and strand 4C, respectively, showed relative insensitivity to the presence of mAb_5E3_ under these conditions, suggesting that they are components of the epitope (Figure 5B, right panel).

## DISCUSSION

**Figure 6:**
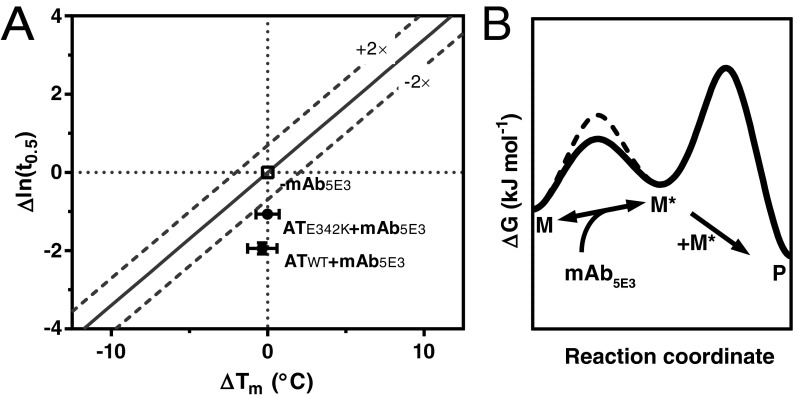
Proposed mechanism for the action of mAb_5E3_ (**A**) A stability-polymerization analysis [10] which compares the average difference in *T*_m_ to the change in the natural logarithm of polymerization half-time. The α_1_-antitrypsin consensus curve determined in that paper is shown as an unbroken line, flanked by dashed lines which represent a 2-fold greater or lesser rate of polymerization than predicted from effects on native state stability alone. Upon binding to mAb_5E3_, the rate of polymerization increases in a manner that is not accounted for by changes in native state stability for AT_WT_ or AT_E342K_ (circles). (**B**) Notional energy landscape in which the presence of mAb_5E3_ facilitates interchange via a stabilized transition state between the native (M) and intermediate (M*) forms, without affecting the stability of either the intermediate or the oligomerization transition state that yields polymer (P).

Effector molecules can be used as tools to probe conformational transitions and thereby provide a means to understand molecular mechanism [21]. In the present study, we have characterized a mAb that increases the rate of polymerization in a dose-dependent manner (Figure 1E), shows a strong preference for polymer over the non-activated monomer (Figures 1B and 1C), does not affect native state stability against change to an activated intermediate (Figure 2B), and does not preferentially act on the barrier between activated monomer and polymer (Figure 3B). However, a stability-polymerization analysis [10] shows that the effect on the polymerization rate for both AT_WT_ and AT_E342K_ variants is greater than expected from negligible effects on native state stability (Figure 6A). Additionally, the antibody interacts with a species that is a hallmark of a polymerization intermediate (Figure 1C), induces polymerization at otherwise non-polymerization-permissive temperatures (Figures 3A and 4A) and increases the rate of polymerization of both the endogenously glycosylated M and Z variants of α_1_-antitrypsin to a level comparable to that of non-glycosylated recombinant protein (Figure 3B). Monovalent interaction by the Fab fragment induces the wild-type variant to behave like that of the Z variant alone (Figure 4B).

With an epitope that appears to include Ser^47^ and Glu^205^ (Figure 5B), this antibody bridges two important regions of α_1_-antitrypsin: Ser^47^ lies adjacent to the Asn^46^ glycosylation site, and Glu^205^ is proximate to Thr^203^, which forms an interaction with Glu^342^, the site of the Z mutation. Given the unexpected coincidence of mAb_5E3_-treated endogenously glycosylated plasma protein with the temperature-dependent rate of polymerization of non-glycosylated recombinant material, it is noteworthy that the Asn^46^ glycosylation site is also implicated in significant effects on altered H/D exchange in the vicinity of Glu^205^ [34].

Collectively, these data are consistent with a mechanism in which mAb_5E3_ acts in two distinct ways: (a) a stabilization of conformational changes that occur in the Ser^47^/Glu^205^ region during transition between the native state and the polymerization intermediate through interaction with a cryptic epitope; and (b) in the context of the full antibody, an increase in the efficiency of the bimolecular association step by optimally orienting the molecules in a manner that reduces the proportion of non-productive interactions resulting from interference by the glycan chains. The latter characteristic is reflected by a lack of templating mechanism and changes in rates of, but not the energetic barrier to, polymerization.

Polymerization is a process that is under kinetic, not thermodynamic, control [10]. Thus, the basis of the susceptibility of the Z variant to polymerization is an increased rate of interchange between–but not the relative stability of–native and activated states [7]. In the context of the subsequent, essentially irreversible, oligomerization step, this increased flux translates overall into an enhanced rate of polymerization. The conformation-stabilizing effect of mAb_5E3_ bears these hallmark characteristics. A mechanism consistent with the cumulative data from the present study is shown in Figure 6(B), in which mAb_5E3_ binds in a reversible fashion to a cryptic epitope that is revealed in the transition state during activation to intermediate, increasing its stability and accordingly reducing the unimolecular activation energy barrier. Against a kinetic backdrop, as with the Z variant, the resulting increased flux translates into an acceleration of the overall polymerization process. In effect, the antibody causes the wild-type M variant to adopt the behaviour of the pathogenic Z mutant.

From this, a number of observations can be made about the polymerization process of α_1_-antitrypsin. The data are consistent with a model in which the presence of glycan chains slow the inter-conversion between native and intermediate conformers, such that mAb_5E3_ exerts a greater effect on glycosylated material. Glycans also increase the proportion of ‘frustrated’ bimolecular interactions, without affecting the energy barrier to polymerization. In addition, the activated state of the Z variant shows a similar amenity to polymerization to that of the wild-type protein, indicating that there are no substantial structural differences induced by the E342K mutation once in this intermediate conformation. Finally, it is of note that the binding of mAb_5E3_ to the intermediate fails to interfere with oligomerization; this indicates that there are structural changes associated with the cryptic epitope that persist from activated monomer to polymer.

In conclusion, we have shown how an antibody raised against a kinetically unstable mutant can induce similar behaviour in the wild-type protein. This monoclonal antibody represents a tool that can be used in further studies to probe the effects of novel mutations and effector molecules on the polymerization intermediate and, in particular, oligomerization.
